# Lessons Learned From Telephone-Based Data Collection for Health and Demographic Surveillance Systems During the COVID-19 Pandemic in Indonesia

**DOI:** 10.9745/GHSP-D-22-00446

**Published:** 2024-04-29

**Authors:** Prima Dhewi Ratrikaningtyas, Lutfan Lazuardi, Agung Nugroho, Amirah Ellyza Wahdi, Rahsunji Intan Nurvitasari, Luthfi Azizatunnisa, Alfianto Hanafiah, Septi Kurnia Lestari, Ratri Kusuma Wardani, Putri Tiara Rosha, Aviria Ermamilia, Fitrina Mahardani Kusumaningrum, Vena Jaladara, Yayuk Hartriyanti, Fatwa Sari Tetra Dewi

**Affiliations:** a Department of Biostatistics, Epidemiology, and Population Health, Faculty of Medicine, Public Health, and Nursing, Universitas Gadjah Mada, Sleman Regency, Indonesia.; b Sleman Health and Demographic Surveillance System, Faculty of Medicine, Public Health, and Nursing, Universitas Gadjah Mada, Sleman Regency, Indonesia.; c Department of Health Policy and Management, Faculty of Medicine, Public Health, and Nursing, Universitas Gadjah Mada, Sleman Regency, Indonesia.; d Nutrition Study Program at University of Aisyiyah Yogyakarta, Sleman Regency, Indonesia.; e Department of Health Behavior, Environment, and Social Medicine, Faculty of Medicine, Public Health, and Nursing, Universitas Gadjah Mada, Sleman Regency, Indonesia.; f Department of Health Nutrition, Faculty of Medicine, Public Health, and Nursing, Universitas Gadjah Mada, Sleman Regency, Indonesia.

## Abstract

The Sleman Health and Demographic Surveillance System (HDSS) is a longitudinal survey held routinely since 2014 to collect demographic, social, and health changes in Sleman Regency, Special Region of Yogyakarta, Indonesia. During the COVID-19 pandemic in Indonesia, we needed to adjust our method of conducting data collection from in-person to telephone interviews. We describe the Sleman HDSS data collection strategy used and the opportunities it presented. First, the Sleman HDSS team completed a feasibility study and adjusted the standard operational procedures to conduct telephone interviews. Then, the Sleman HDSS team collected data via a telephone interview in September–October 2020. Ten interviewers were equipped with an e-HDSS data collection application installed on an Android-based tablet to collect data. The sample targeted was 5,064 households. The telephone-based data collection successfully interviewed 1,674 households (33% response rate) in 17 subdistricts. We changed the data collection strategy so that the Sleman HDSS could still be conducted and we could get the latest data from the population. Compared to in-person interviewing, data collection via telephone was sufficiently practical. The telephone interview was a safe and viable data collection method. To increase the response rate, telephone number activation could be checked, ways of building rapport could be improved, and engagement could be improved by using social capital.

## Abstract (precis)

We document valuable experiences and challenges when collecting data for a routine longitudinal survey using telephones instead of in-person interviews during the pandemic.

## Teaser

**Key Findings:**

- During the pandemic, conducting telephone-based interviews allowed data to be collected for the routine longitudinal survey safely without direct respondent contact and without interruption.
- Establishing trust, encouraging social norms, describing the reciprocal benefits of survey participation, and leveraging social networks facilitated survey participation and data collection.
- Despite using strategies to encourage survey responses, the response rate for telephone-based data collection was lower than in-person data collection.

**Key Implications:**

- Before conducting a telephone-based interview, researchers should conduct a feasibility study to explore the respondents’ characteristics, including the cost impact on respondents, respondents’ telephone ownership, and capabilities to interact by telephone (limited for the older age or respondents with disabilities), as well as connection strength and interview length.
- A longitudinal study in Indonesia should collect respondent contact numbers and update them frequently to ensure they are still active.

## BACKGROUND

As the demand for health care services rises, interest in longitudinal population studies is increasing. A longitudinal population study is valuable to identify trends in health services, factors related to health services, and their impact on improving physical and mental health.^1^ Previous studies established the importance of population-based longitudinal studies to monitor the COVID-19 pandemic and its effects.^2^

The Sleman Health and Demographic Surveillance System (HDSS) is a longitudinal survey system that collects data on demographics and social and health status changes in Sleman Regency, Special Region of Yogyakarta, Indonesia. Sleman Regency (Figure) represents both urban and rural areas of Indonesia. The population of this regency has a relatively high life expectancy and a high prevalence of noncommunicable diseases and their risk factors. The Sleman HDSS aims to collect high-quality data as the basis for planning educational activities and policymaking to improve the population’s health quality. The Sleman HDSS used a quantitative method that involved 5,147 households in 216 clusters (184 urban and 32 rural) in 80 selected villages in Sleman Regency. Details regarding the Sleman HDSS design are available elsewhere.^3^

**FIGURE:**
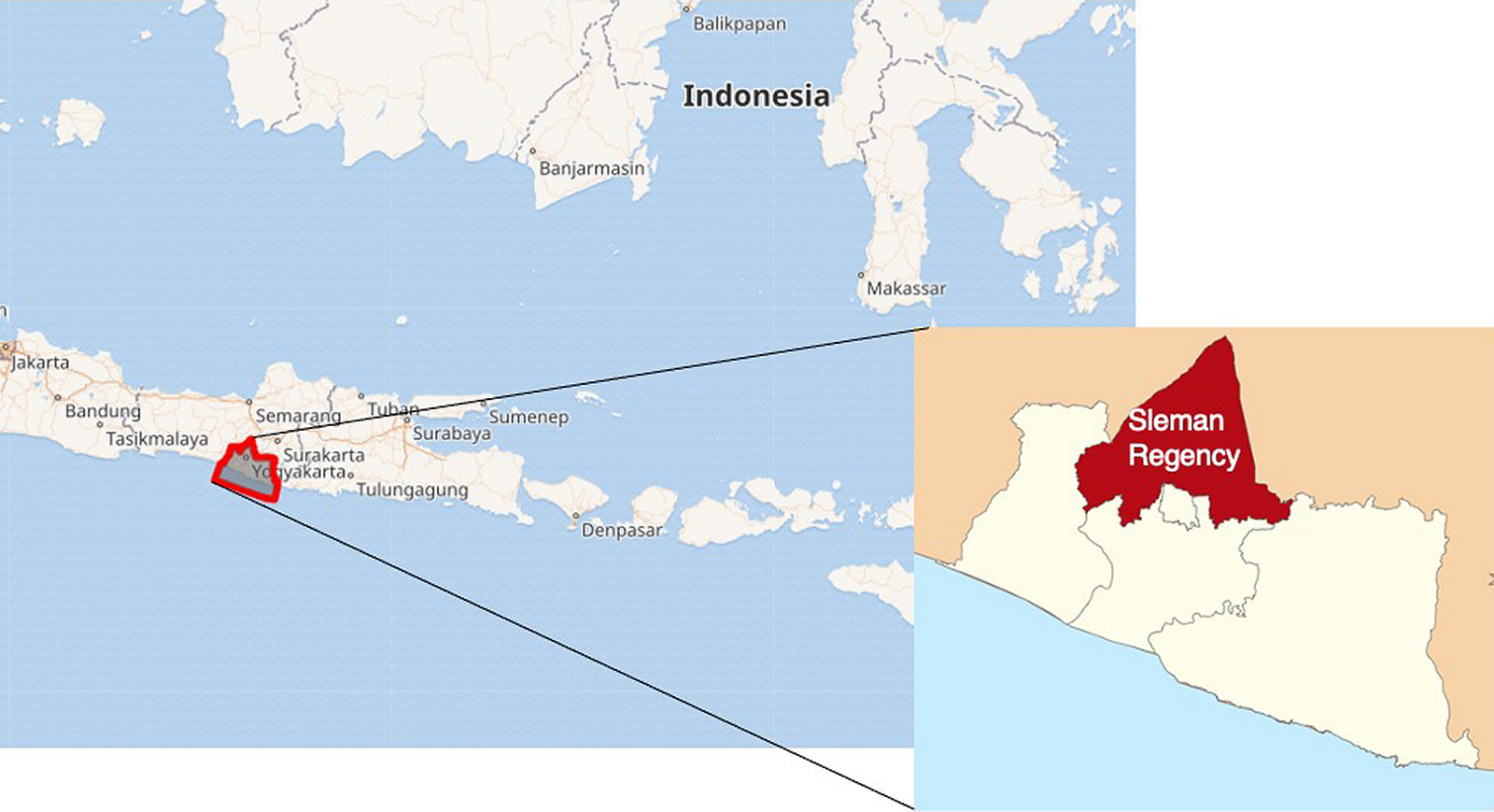
Map of Sleman Regency, Special Region of Yogyakarta, Indonesia **Source**: © OpenStreetMap Contributor.

In March 2020, the Director-General of the World Health Organization declared COVID-19 a global pandemic.^4^ On March 2, 2020, the Indonesia government announced the first COVID-19 case, prompting the government to stop COVID-19 transmission by closing schools and offices, limiting religious activities, and restricting any activities in public spaces or facilities, including in-person data collection.^5^

Data collection during a pandemic is essential to monitor the impact of COVID-19 on the economic sector,^6^^,^^7^ health care service access,^8^^,^^9^ changes in health-related behaviors,^10^ and mental health.^11^^–^^13^ The Sleman HDSS team designed alternative strategies for collecting data during the pandemic. One of the proposed strategies was replacing in-person interviews with telephone interviews. Several large-scale household surveys have been conducted via telephone interviewing^14^^,^^15^ because it enables high-frequency data collection in a shorter time and is more cost effective.^16^^,^^17^ Based on a previous study that surveyed household information technology utilization patterns at Sleman Regency in 2019 (400 households), 92.75% owned cellular phones, 7.29% owned cable telephones, and 49.75% of respondents used the Internet to access social media applications, such as WhatsApp and Instagram.^18^ Telephone-based data collection can collect valid and reliable data.^16^^,^^17^^,^^19^ However, when designing telephone-based data collection, several factors need to be considered, including contact availability,^17^^,^^20^ completion, response rate, demographic representative,^20^ costs,^14^ data consistency, and bias minimization.^17^ We document the Sleman HDSS team’s experience in conducting the wave 6 survey during the pandemic using telephone-based data collection in 2020 and share lessons learned. We describe the processes and activities that were adjusted during the pandemic, including preparation, data collection process, and data management.

Telephone interviewing enables high-frequency data collection in a shorter time and is more cost effective.

## PREPARATION

We conducted a feasibility study to explore HDSS respondents’ perceptions of data collection via telephone using a qualitative method with a rapid assessment procedure. Twenty-four respondents were interviewed in depth via telephone, including households, heads of the village, and researchers with experience in conducting telephone interviews. Results revealed that respondents preferred in-person interviews over telephone interviews. However, the pandemic conditions did not give us much choice in data collection methods. A telephone interview could be implemented by considering several factors, including connection strength, interview time and duration, cost impact to respondents, respondents’ telephone ownership, and the ability to communicate by telephone. Results showed that the respondents expected interviews to be conducted in their free time and last about 20 to 30 minutes.

Initial contact with respondents was a crucial part of engaging with them. The feasibility study results suggested sending a short message that included standardized study information and asking for the respondent’s willingness to participate in this study. During the initial contact with respondents, the interviewer’s credentials were proved by displaying the Sleman HDSS attributes in the interviewer’s contact profile and showing a letter of assignment. Several respondents’ contact numbers in the Sleman HDSS database were invalid or inactive. As a result, the feasibility study results recommended checking the respondents’ numbers with community stakeholders.

Then, we developed a standard operating procedure (SOP) to collect data via telephone. In the adjusted SOP, the initial contact had to ensure that the telephone number called was active and was indeed the number of the Sleman HDSS respondent’s household members. In addition, the Sleman HDSS team adjusted the SOP for distributing an interview incentive (telephone or Internet credits/e-money) and changed the informed consent to verbal informed consent. Before starting the interview, we explained the purpose and methods of our research to all the participants. We also asked them if they were willing to have their voices recorded during the interview. Only those who gave their consent were recorded, and we repeated the consent questions at the beginning of the recording as a confirmation.

We prepared the questionnaire and data collection application using the hybrid e-HDSS, an electronic-based questionnaire using a mobile device.^3^ This application has several features, including the ability to record voice, automatically show questions that meet the predetermined rule (e.g., respondent characteristics and question skipping pattern), automatically generate identity numbers, and recall basic demographic data from the Sleman HDSS database.

### Ethical Approval

The verbally informed consent procedure for the Sleman HDSS was approved by the Medical and Health Research Ethics Commission, Faculty of Medicine, Public Health, and Nursing, Universitas Gadjah Mada (approval number KE/FK/0586/EC/2020.)

## DATA COLLECTION PROCESS

We collected data in September–October 2020. Usually, the Sleman HDSS team would request permission for data collection by visiting the local leaders (the subdistrict head office, head of the village, and head of the subvillage). However, due to the COVID-19 restrictions, data collection permission was requested by mail and by contacting the local leaders via telephone or WhatsApp. We divided the interview process into several stages: initial contact, interview consent request, household module interview, and individual module interview.

Initial contact aimed to confirm that the telephone number was active and owned by the Sleman HDSS respondent. The interviewer contacted the respondents in the interview list through text messages or WhatsApp messages to explain the aim of the interview. Afterward, the interviewer confirmed the potential respondents’ identities by requesting the respondents’ full names and addresses. If both name and address data matched, the interviewer explained the survey procedure and confirmed the respondents’ willingness to participate. If the name and address did not match, the interviewer would check with the database and report it as the wrong number.

Research information and requests for interview consent were delivered verbally or through text/chat by the interviewer. Confirmation of respondents’ identity, verbal informed consent, and respondents’ statement of consent were recorded as proof of respondents’ participation in this study. Subsequently, the interviewer would send text reminders concerning the data collection time and confirm the preferred communication mode. After we confirmed the respondent’s identity and received consent, we proceeded to the interview stage.

For the household question module, 1,674 households were successfully interviewed, and for the individual question module, 1,525 respondents were successfully interviewed. The main reasons for the initial failed contact were an inactive number (66%), participants who did not respond (26%), and misdialed numbers (5%). The strategy used to minimize interview failures involved contacting the respondent at least 3 times at different times, contacting other family members, and asking for help from community leaders (e.g., head of the subvillage) or the health cadre to inform the respondent concerned.

The response rates for household and individual question modules were 33%.

There are 1,665 rows in the original data recapitulation for wave 6, but in the audio file processing, only 1,644 rows are involved. This is due to 19 recordings excluding interview time and 2 recordings containing unanswered calls. Table 1 shows the information and duration of interviewed data collection by telephone. The mean duration of the interview was 31.73 minutes (standard deviation: 43.13 minutes). Most respondents were successfully interviewed only once (90.39%), and the most frequently chosen time for the interview was afternoon (43.67%).

**TABLE 1.** Summary of Telephone Interview Results

**Variables**	**Total Respondents, No. (%) (n=1,644)**
Interview frequency per respondent	
Once	1,486 (90.39)
More than once	158 (9.61)
Time of interview	
Morning	342 (20.80)
Noon	718 (43.67)
Afternoon	584 (35.52)
Duration of interview	
Less than 20 minutes	348 (21.17)
20–30 minutes	518 (31.51)
More than 30 minutes	778 (47.32)

Table 2 compares the Sleman HDSS respondents’ characteristics in the fifth wave in 2019 (in-person interview), sixth wave in 2020 (telephone interview), and seventh wave in 2021 (in-person interview). The proportion of household wealth status, level of education, current employment, number of household members, and head of household’s age were significantly different between waves.

**TABLE 2.** Comparison of the Sleman HDSS Respondents’ Characteristics Between In-Person Interviews (Fifth and Seventh Waves) and Phone-Based Interviews (Sixth Wave)

**Characteristics**	**Wave 5 (In-Person), No. (%) (n=4,889)**	**Wave 6 (Phone-Based), No. (%) (n=1,674)**	**Wave 7** **(In-Person), No. (%)** **(n=4,529)**	***P* Value** ^a^
Household wealth index				.000
Low	1,945 (40.53)	497 (30.72)	1,733 (40.72)	
Medium	1,999 (41.65)	711 (43.94)	1,820 (42.76)	
High	855 (17.82)	410 (25.34)	703 (16.52)	
Homeownership status				.244
Private property	3,418 (72.52)	1,120 (70.48)	3,085 (72.50)	
Rent/other	1,295 (27.48)	469 (29.52)	1,295 (27.50)	
Head of household age				.000
Less than 20 years	2 (0.04)	0 (0.00)	1 (0.02)	
Early adulthood (20–40 years)	619 (12.66)	221 (13.20)	470 (10.38)	
Middle adulthood (41–60 years)	2,596 (53.10)	978 (58.42)	2,376 (52.47)	
Late adulthood (>60 years)	1,672 (34.20)	475 (28.38)	1,681 (37.12)	
Head of household educational level				.000
Low (no education and elementary school)	1,570 (32.20)	363 (21.72)	1,408 (31.09)	
Middle (junior and senior high school)	2,594 (53.20)	969 (57.99)	2,488 (54.93)	
High (diploma and higher education)	712 (14.60)	339 (20.29)	633 (13.98)	
Head of household employment				.001
Stay-at-home parent and student	176 (3.60)	60 (3.58)	210 (4.64)	
Unemployed	499 (10.21)	154 (9.20)	511 (11.28)	
Employees and workers	2,050 (41.93)	740 (44.21)	1,877 (41.44)	
Retired	466 (9.53)	169 (10.10)	434 (9.78)	
Entrepreneur	858 (17.55)	308 (18.40)	750 (16.56)	
Service	219 (4.48)	74 (4.42)	188 (4.15)	
Farmer-livestock	571 (11.68)	141 (8.42)	480 (10.60)	
Other	50 (1.02)	28 (1.67)	70 (1.55)	
Region				.231
Urban	4,048 (82.80)	1,414 (84.47)	3,757 (82.95)	
Rural	841 (17.20)	260 (15.53)	772 (17.05)	
Number of household members				.000
1–2 people	862 (17.63)	234 (13.98)	800 (17.66)	
3–5 people	3,175 (64.94)	1,150 (68.70)	2,865 (63.26)	
>5 people	852 (17.43)	290 (17.32)	864 (19.08)	
Communicable diseases modules				
Dengue fever	67 (0.35)	15 (0.22)	19 (0.11)	
Crude birth rate^b^	7.85	11.3	6.99	
Crude death rate^b^	0.008	0.010	0.010	

Abbreviations: HDSS, Health and Demographic Surveillance System.

^a^ Chi-square test.

^b^ Per 1,000 persons in a population.

## DATA MANAGEMENT

We ensured that the quality of data collected met the standard by using the e-HDSS and conducting spot-checking, cross-checking, and data cleaning. The spot-checking process ensured the data collection process followed the SOP. Before the pandemic, supervisors conducted spot-checking by observing interviewers during in-person interviews in respondents’ houses and then recording their observations on the form provided. During the COVID-19 pandemic, supervisors conducted spot-checking by listening to interview recordings. They performed spot checks at the beginning of the data collection process (first week of September) and in the middle of data collection (first week of October). To check for any misses, matching was conducted to see the compatibility between the respondent’s answers and the data entered by the interviewers into the questionnaire.

The cross-checking procedure involved listening to the interview recording and matching the recording with the data uploaded to the Sleman HDSS server. Around 5% of household interview recordings were randomly selected for cross-checking.

There was no difference in data-cleaning methods before and after the pandemic. The Sleman HDSS data manager handled data cleaning from initial contact reports and daily interview reports and cleaned the interview data, including checking data completion and validation rules, double-checking data with previous wave data, and tabulating data. The cleaning process aimed to find any errors in the questionnaire filling process. Based on the cleaning process result, the interviewers were asked to confirm any errors, using the interview recordings and field notes for correction. After the data were cleaned and met the established standard, the data manager would release the dataset for the data analysis process. The Sleman HDSS sixth wave data were released on July 17, 2021.

## BENEFITS OF TELEPHONE INTERVIEWS

### No Direct Contact With Respondents

Data collection using the telephone could be done without making physical contact with respondents, so it was very appropriate to use during the pandemic.^21^ In response to future pandemic events, data collection by telephone can be used and can complement in-person interview data collection.^22^

### No Disruption of Routine Survey

The COVID-19 pandemic affected the lives of many people in low- and middle-income countries, including Indonesia. During the pandemic, many activities were hindered, including activities that involved community participation. Despite these challenges, we were still able to conduct the Sleman HDSS during the pandemic. To understand the impact of the pandemic on the population, the survey collected data from households and individuals. For individuals, we focused on collecting information on respondents’ socioeconomic status, health access, mental well-being, physical activity, and smoking behavior. We recognized that household-level data may not have been representative of the whole population, so we also analyzed the individual-level data to capture the variations among different individuals.

## LESSONS LEARNED

### Social Capital Facilitated Survey Implementation

Social capital is defined as trust, norms, reciprocity, and networks between individuals.^23^ Taking advantage of existing social capital facilitated the implementation of the survey.

Respondents’ participation was indirectly influenced by trust in Universitas Gadjah Mada (UGM), the oldest state university in Indonesia. When the respondent knew that the survey was conducted by UGM, the respondent responded well. UGM’s credibility made respondents believe that this survey could be trusted and was useful for the community. Because the Sleman HDSS is longitudinal, it requires long-term collaboration with the community. Thus, HDSS routinely held activities, such as community service activities with HDSS researchers and UGM lecturers, to familiarize and strengthen bonds with respondents.

Respondents’ willingness to participate was influences by trust in the survey team, social norms, and reciprocity between the survey team and respondents.

Social norms were evident when respondents were reluctant to refuse the interview for fear of being considered disrespectful after getting permission from the hamlet or local officials. In addition, interviewers were asked to maintain politeness, speak at a calm tempo, and add sufficient “small talk” to maintain rapport, even when there was no in-person interaction.

During the informed consent process, the Sleman HDSS team explained that the survey would contribute to the health sector, especially in the Sleman area, so respondents felt that there would be reciprocity for their participation and were then willing to participate in the survey.

Telephone data collection used social networks owned by the Sleman HDSS team that started by contacting or communicating to the subdistrict, village, or hamlet head to ask for the respondent’s phone number.

### Lack of Availability of Respondents’ Contact Number Hindered Data Collection

One of the significant challenges in conducting telephone interviews was the lack of availability of respondents’ contact numbers. Of 5,604 households, only 3,676 (72.59%) had their contact numbers stored in the HDSS database. The Sleman HDSS team also requested aid from local community figures to provide an additional 436 household contact numbers. Based on the Regulation of Communication and Informatics Minister of Indonesia, No. 14 of the Year 2017 on Telecommunication Service Customer Regulation, each customer can only register a maximum of 3 different numbers for every Citizenship Identity Number.^24^ However, many customers own over 3 different numbers and frequently change numbers, resulting in difficulty for Sleman HDSS to track which number is currently active.^25^

### Alternative Strategies Should Be Pursued to Improve Response Rate

Data collection via telephone was viable for the Sleman HDSS respondents despite the challenges and lower response rate. The response rate was influenced by respondents being contactable via telephone and the respondent’s willingness to participate.^14^ The Sleman HDSS team designed various strategies to increase the response rate, such as adjusting the call time according to the respondent’s profession, calling back up to 5 times, and sending reminder messages through WhatsApp. Subsequent studies are needed to investigate different methods to improve response rates from respondents with active numbers but who did not respond.

Subsequent studies are needed to investigate different methods to improve response rates from respondents with active numbers but who did not respond (the nonresponse rate was about 15%). Despite a low response rate, telephone-based data collection generally shows little bias from nonresponse on lifestyle, health, and demographic questions.^26^ A detailed analysis of the relationship between nonresponse rates and nonresponse bias from 30 published studies found that the response rate alone was not a very good indicator of the magnitude of nonresponse bias.^27^^,^^28^

### Respondents’ Telephone Literacy Limited Participation

The percentage of the population using cellular phones continued to increase until 2019, reaching 63.53%.^29^ Social media dominates the purpose of Internet use (87.20%).^29^ Using cell phones to take online surveys is not yet common in Indonesian society. Furthermore, because respondents were not met in person for the survey, it was easier for them to refuse/ignore telephone messages to participate. Data collection via telephone requires adequate technical skills and preparation in using the telephone for both the researcher and the respondent.^30^

## CONCLUSIONS AND RECOMMENDATIONS

COVID-19 prompted a shift in data collection methods for population surveys from in-person interviews to telephone-based interviews, creating many opportunities that can be learned from conducting surveys elsewhere. This method was sufficiently safe to be conducted during the pandemic. We gained much information about the limitations of telephone surveys and how to overcome them, which could also help improve our research strategies in the post-pandemic era. Social capital and improvement in telephone literacy could help increase the response rate for data collection by telephone.

## Notes

**Peer Reviewed**

**First published online:** March 4, 2024.
